# Design and evaluation of vaccines for the control of the etiological agent of East Coast fever

**DOI:** 10.1186/s13071-024-06517-w

**Published:** 2024-11-20

**Authors:** José de la Fuente, Isidro Sobrino, Margarita Villar

**Affiliations:** 1 SaBio, Instituto de Investigación en Recursos Cinegéticos IREC-CSIC-UCLM-JCCM, Ronda de Toledo 12, 13005 Ciudad Real, Spain; 2 Department of Veterinary Pathobiology, Center for Veterinary Health Sciences, Oklahoma State University, Stillwater, OK 74078 USA; 3 Biochemistry Section, Faculty of Science and Chemical Technologies, University of Castilla-La Mancha, 13071 Ciudad Real, Spain

**Keywords:** East Coast fever, Tick, Tick-borne diseases, Vaccine

## Abstract

East Coast fever is a tick-borne theileriosis caused by *Theileria parva*, a protozoan parasite with the primary vector being the tick *Rhipicephalus appendiculatus*. This disease poses significant challenges in sub-Saharan Africa, leading to severe economic losses by causing the death of over one million livestock annually. Current control measures include vector control with acaricides and the “infection and treatment” method, which involves immunization with live sporozoites of the pathogen and treatment with long acting oxytetracycline. Despite their effectiveness, these methods face scalability and usability issues, necessitating the development of new prevention strategies, particularly in the field of vaccines for the effective and sustainable control of East Coast fever. In this primer focus, East Coast fever serves as a case study to highlight recent concepts and advancements in tick and tick-borne disease vaccine research. Vaccine design and evaluation processes are reviewed, encompassing the utilization of omics datasets and knowledge on vectors and pathogens, and exploring new design methods, such as quantum vaccinomics and messenger RNA (mRNA)-based vaccines. Key limitations and areas requiring further research are addressed, including insufficient understanding of host–pathogen molecular interactions, the impact of post-translational modifications, and vaccine efficacy variability across different trials. Additionally, new research objectives are proposed to address East Coast fever but with possible impact on other tick-borne diseases. It includes advancing knowledge on tick–pathogen–host molecular interactions, studying tick microbiota, developing novel design approaches, such as combining tick and pathogen epitopes in chimeric vaccines (exemplified by the q38–p67c case), and exploring new immunological enhancers and delivery platforms.

**Graphical Abstract:**

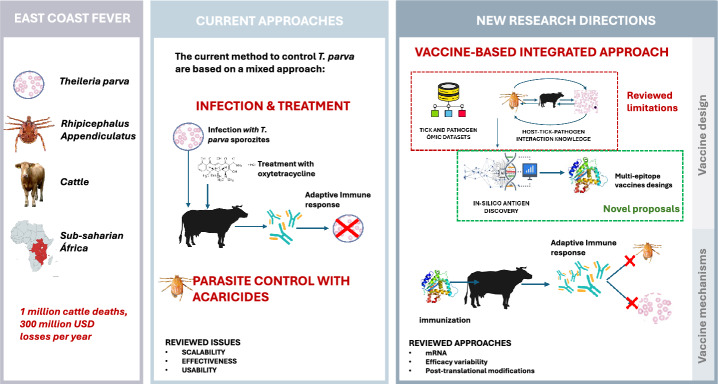

## Background

Tick-borne diseases constitute a global threat to human and animal health [1–3]. Integrated control strategies with One Health approach combining rational application of acaracides, pharmaceuticals and vaccines with animal management is the most effective strategy for the control of tick-borne diseases [4, 5]. As reviewed in ref. [6], vaccines offer effective and sustainable intervention for disease control and for tick-borne diseases targeting both vectors and transmitted pathogens. Applying a personalized vaccinology approach considering host-tick-pathogen genetic factors and international collaborations are important with emphasis on developing countries [7]. The combination of vector and pathogen derived antigens in vaccine formulations is a challenge which has potential to reduce tick infestations while boosting protective immune response against pathogen infection [8].

As an example, herein, we will use East Coast fever (ECF) to evaluate possibilities for the design and evaluation of vaccines to control this and other tick-borne diseases. East coast fever, also known as theileriosis, is a tick-borne disease that is caused by the Piroplasmida organism, *Theileria parva* [9–11]. The main vector for *T*. *parva* is *Rhipicephalus appendiculatus* ticks with transmission from persistently infected cattle even in the absence of detectable parasitemia [10]. Usually, *T*. *parva* acquired by feeding ticks mature to the infection form and transmitted via saliva after ectoparasite attachment to the host without transovarial transmission. In the host, *Theileria* sporozoites life cycle include replication of schizonts in leukocytes and piroplasms in erythrocytes [10, 11].

The disease has a major economic impact on cattle health and production in sub-Saharan Africa, where it is associated with high fever, diarrhea, dyspnea, eye and nose mucous discharge, and the death of over one million cattle every year, with the total economic impact estimated at over 300 million USD as recently reviewed [12–15]. Currently, as reviewed in [9, 16], the control of this disease is implemented by minimizing the risk of transmission and/or by reducing tick vector populations using chemical acaricides. An antiprotozoal treatment, buparvaquone, can also be used to treat infected cattle [9, 16]. However, the effectiveness and long-term efficacy of these approaches can be reduced by the selection of acaricide-resistant tick vectors and buparvaquone-resistant related cattle pathogen, *Theileria annulata*.

## Interventions for the prevention and control of East Coast fever

East Coast fever presents usual clinical signs including swelling of the draining lymph node followed by lymphadenopathy, fever, petechial and ecchymotic hemorrhage on conjunctiva and buccal cavity mucous membranes, and anorexia. Other clinical signs may include lacrimation, nasal discharge, corneal opacity, terminal dyspnea and diarrhea [11]. As recently reviewed, the prevention of ECF relies on a strategy referred to as “infection and treatment method” (ITM), which is the simultaneous infection with live *T*. *parva* sporozoites and treatment with the long-lasting antimicrobial oxytetracycline [9, 16]. While ITM can be highly effective, it has several limitations, including the consistency and scalability of manufacturing process, the requirement for cold-storage chains, and geographic-based antigenic diversity in *T*. *parva* strains [17]. As ITM elicits immunological responses that provide long-term protection from ECF, it supports that a safe and effective vaccine would improve the control of this disease [9, 16].

For vaccine development, the major surface antigen p67 of *T*. *parva* was identified with the capacity for the development of an effective ECF vaccine [18]. The p67 is a major sporozoite surface protein possibly involved in attachment and infection of host lymphocytes [19]. Studies suggested that two discontinuous epitopes within an 80 amino acid domain from the C-terminus of p67 containing protective epitopes and referred to as p67C, induce protective immune responses [20]. The results showed that three doses of p67C provided a 50% efficacy at 70% lethal dose (LD70) to cattle from ECF challenge like the whole p67 antigen [20]. However, reducing the number of immunizations to two resulted in a dramatic loss of protective efficacy to 25% at LD70 [21]. Antibody and CD4 T-cell responses to p67C were observed in cattle following co-immunization with 70–80 μg p67C per dose delivered via two nanoparticle technologies with 53% vaccine efficacy at LD93 [22]. Recently, a vaccine efficacy close to 50% against sporozoite challenge was reported with 140 μg p67C per dose delivered using a computationally designed nanoparticle [23]. Additionally, although buffalo-derived strains have shown p67 genetic diversity [24], in this study p67 showed 100% identity in different field strain genotypes of *T*. *parva*, thus suggesting protective capacity against different strains [20]. Altogether, these results support that p67 is a good candidate protective antigen for vaccine development against *T*. *parva*, but vaccine trials should include challenge under natural conditions and not only highly artificial needle challenge to address the question if p67C alone can be sufficient to develop a new generation vaccine or will be additional antigenic sequences needed.

## Gaps in the characterization of host–pathogen and tick-pathogen molecular interactions

Some aspects of *T*. *parva* biology with knowledge gaps need to be considered for development of vaccines and other control interventions as reviewed in ref. [25]. The first gap is the poor understanding of host and tick cell infection and the potential importance that post-translational modifications of pathogen proteins may play in this process. The recent identification of components of N-glycosylation in *T*. *parva* RNAseq datasets suggest the possibility that this parasite can produce glycosylation of proteins and lipids as integral components for the attachment and infection process of Apicomplexan parasites [26, 27].

The second gap is the information regarding *T*. *parva* and *R*. *appendiculatus* transcriptome and proteome throughout life cycle to select candidate protective antigens [25]. The available omics datasets for *Theileria* and *Rhipicephalus* is limited (Table 1) and this information is important for the identification of candidate protective antigens. Recently, new candidate protective antigens were identified using comparative transcriptomes and bioinformatic data analyses [28]. Then, two candidate antigens encoded by *T*. *parva* strain Muguga genes TP04_0076 (https://www.ncbi.nlm.nih.gov/gene/?term=TP04_0076) and TP04_0640 (https://www.ncbi.nlm.nih.gov/gene/?term=TP04_0640) coding for putative integral membrane and PIG-P family proteins, respectively, were selected for validation studies. These genes were likely to encode membrane proteins and were predicted to have high antigen-presenting and immunogenic capacity to induce humoral and/or cell-mediated immune response. The antigenicity of the encoded polypeptides was confirmed using a bacterial-expressed polypeptide (TP04_0076) and synthesized peptides (one for TP04_0076 and two TP04_0640) with serum samples from cattle positive to *T*. *parva* [28]. However, these antigens have not been evaluated in cattle for protective capacity against pathogen infection. The identification of protective antigens/epitopes considering pathogen and host derived genetic factors is crucial to advance in vaccine development.

**Table 1:** Available omic datasets for *Theileria* and *Rhipicephalus* species

Species	Genome level	DNA	RNA	Bio	Protein	CDP	PRIDE databases
*T*. *parva*	Chr	1864	8408	20	2787	Close to standard (low value)	0
*Theileria* spp.	Complete (for some spp.)	13,582	67,301	52	30,286	Standard (for some spp.)	3
*R*. *appendiculatus*	Contig	576	19,279	6	12,922	No proteome	0
*Rhipicephalus* spp.	Chr (for some spp.)	314,330	68,380	132	12,022	Close to standard (high value) (for some spp.)	10

Data (accessed on 23 June 2024) is disclosed for ECF pathogen and main vector (*T*. *parva* and *R*. *appendiculatus*) but also for other *Theileria* and *Rhipicephalus* spp. as it is relevant for omics analyses

Chr: chromosome; DNA and RNA: number of sequences from GenBank (https://www.ncbi.nlm.nih.gov/genbank/); Bio: deposited datasets from BioProject (https://www.ncbi.nlm.nih.gov/bioproject); Protein: number of proteins from Uniprot (https://www.uniprot.org); CDP: proteome database coverage (https://www.uniprot.org); PRIDE: proteomics identification database (https://www.ebi.ac.uk/pride/)

The third gap is the limited information of receptors and *T*. *parva* ligands involved in tick-pathogen interactions and infection. The identification of these proteins may provide new targets to reduce pathogen infection and transmission by *R*. *appendiculatus* tick vector using vaccines and paratransgenic interventions [29].

The fourth gap is the poor understanding of what constitutes a protective immune response against pathogen infection [25]. It is generally accepted that strong cell-mediated responses play crucial roles in providing protection from and reducing the severity of *T*. *parva*-associated disease. It has been shown CD8 T-cell mediated immune response to protective epitopes in antigens Tp1 and Tp2 (e.g., [30]). However, the mechanisms of protective immune response to vaccination need further characterization and the complexity and risk profiles of *T*. *parva* associated with variations in the challenge sporozoite dose and/or natural host susceptibility to infection should be considered. These variables may impact the vaccine efficacy estimates, making it difficult to compare between trials over time. Accordingly, improved knowledge of protective immune response can be used to complement in vivo challenges with in vitro assays. This approach also has the capacity to advance knowledge and improve animal welfare by experimental designs where only animals with optimal immune responses are challenged and are therefore likely to be protected.

## Recent advances in vaccinology

Novel vaccinology platforms and algorithms are required to further advance in the development of vaccines for the control of cattle tick infestations and tick-borne diseases [29]. Recent algorithms led to quantum vaccinomics for the development of vaccines and other control interventions [29]. These novel interventions include (a) use of commensal bacteria to produce and secrete protective antigens to interfere with pathogen infection or serve for vaccine delivery, (b) combination of vaccines with probiotics (e.g., with high alpha-gal content) and heat inactivated mycobacteria to serve as adjuvants/immunostimulants, (c) new vaccine delivery platforms [e.g., nanoparticle (NP)-based formulations, lipid NP-mRNA, viral vectors, virus-like particles (VLPs)] to stimulate innate and trained immunity and boost protective immune response, (d) and oral vaccine formulations to improve safety and access to developing countries [29, 31, 32].

Even when protective antigens are identified or designed, formulations and delivery platforms are key components of vaccine efficacy associated with induced type and duration of immune response [6]. Regarding tick control, recent advances in vaccine formulations targeting vector gut microbiota commensal bacteria was found effective [33]. Experimental microbiota manipulation has been achieved by antibiotic exposure or sterile-rearing conditions of the vector. Anti-microbiota vaccine impacts tick physiology and pathogen infection by modulated tick microbiota composition and diversity [33, 34]. However, these methods induce global changes in the microbiota and make the depletion of specific bacteria difficult. Other advances include probiotics and formulations with high alpha-gal content [35] and adjuvants with heat-inactivated alpha-gal-containing bacteria for oral vaccine administration [31]. For example, oral vaccine formulations combining *R*. *appendiculatus*-derived SUB with heat-inactivated mycobacteria resulted in 96% and 99% efficacy (i.e., reduction in tick infestations, feeding, and reproduction) against *R. decoloratus* and *R*. *appendiculatus*, respectively [31].

The mRNA vaccines have the potential to address the possibility for protein antigens to have post-translational modifications, such as glycosylation [9]. With an appropriate design, the expressed antigen can be directed to the endoplasmic reticulum to undergo the addition of glycan moieties. Clearly, the capacity of *T*. *parva* to complete the post-translational modification of potential antigens will need to be confirmed before candidate antigens are directed into these pathways. Furthermore, some post-translational modifications, such as the glycosylation of cryptic motifs in the pathogen polypeptide might mask or sterically hinder the recognition of protective epitopes and should be considered.

The combination of protective antigens may be used to improve vaccine efficacy. However, combining antigens in vaccine formulations requires innovative approaches to prevent antigen competitions with negative effect on host protective immunity [29, 36]. Antigen competition is based on reciprocal, humoral, cellular, and intermolecular type responses with partial or complete suppression of immune response against one antigen that resulted from stimulation with a second unrelated antigen or the recognition of non-protective epitopes by some immunized hosts. Quantum vaccinomics is an innovative approach for the design of new protective antigens by combining protective epitopes or immunological quantum in a chimeric antigen. Multiple methodological approaches on the basis of the characterization of protein–protein interactions and their role on immune response have been developed for quantum vaccinomics [29]. Then, vaccine formulations based on chimeric antigens combining the identified protective epitopes may be used to boost protective immune response against multiple antigens [37].

## Combination of tick with pathogen derived antigens

As an example, the combination of tick protective antigens was approached with Subolesin/Akirin protective epitopes chimera Q38 [37] and P67C (P67 amino acids 572–651) [38]. Sequence alignment of Q38 tick chimeric antigen and *R*. *appendiculatus* ABA62331.1 protective antigen 4D8, also known as Subolesin showed an 84.4% sequence identity in protective epitopes using CLUSTAL 2.1 multiple sequence alignment (https://www.genome.jp/tools-bin/clustalw), thus supporting conservation of Subolesin protective epitopes in the target tick species (Table 2). Nevertheless, changes in amino acids could be introduced to better fit local pathogen genotypes applying a personalized vaccinology approach.

**Table 2:** Sequence alignment of Q38 tick chimeric antigen protective antigen 4D8, also known as Subolesin

Sequence Q38 (NH_2_-COOH)
MACATLKRTHDWDPLHSPNGRSPKPSPFGEVPPKSSPLESGSPSATPPASPTGLSPGGLLSPVRRDOPLFTFRQVGLICERMMKERESQIRDEYDHVLSAKLAEQYDTFVKFTYDQIOKREEGATPSYLSGGGSHKPFGSPSSPSSSAIAAAAAAAKRPSPFAEAVCPKOLTFNTGSRPDSPPSMVLFTFKQALREQYDAVLTNKLAEQYDAAAPSYLS
ABA62331.1 protective antigen 4D8 [*Rhipicephalus appendiculatus*] (NH_2_-COOH)
MACATLKRTHDWDPLHSPSGRSPKRRRCMPLSPPPTRAHQIDPSPFGDVPPKLTSEEIAANIREEMRRLQRRKQLCFQGTDAESQHTSGLSSPVRRDQPLFTFRQVGLICERMMKERESKIREEYDHVLSTKLAEQYDTFVKFTYDQIQKRFEGATPSYLS
Q38-4D8 sequence alignment


Sequence alignment was conducted using CLUSTALW 2.1. (https://www.genome.jp/tools-bin/clustalw) with Score1219 and Alignment Score 596

**Fig. 1:**
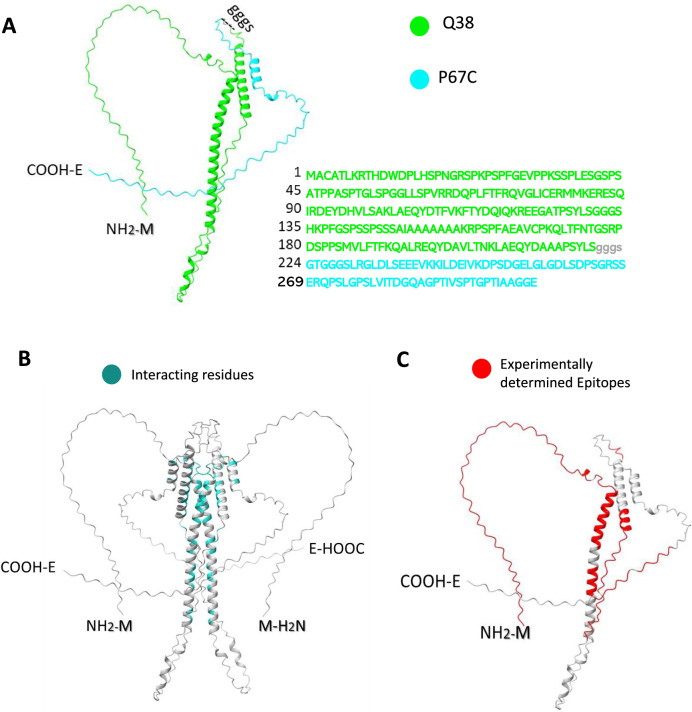
Modelling Q38-P67C chimeric protein, homodimer interactions, and predicted B-cell epitopes. **A** The protein structure was modeled using Alphafold3 (https://golgi.sandbox.google.com/), Q38 residues in green and P67C residues in blue. **B** Dimeric structure and interactions of Q38-P67C (interacting residues in dark green) were modeled using Alphafold3 (https://golgi.sandbox.google.com/) and the interaction was confirmed by the Local Interaction Score (https://github.com/flyark/AFM-LIS). **C** Experimentally determined linear and conformational B-cell epitopes (red residues) showed no significant overlap between epitopes and interacting residues. Epitopes were determined by experimental methods in previous studies [41, 42]

The Q38-P67C chimeric antigen was design and structure modeled (Fig. 1). The results showed helix and strand-coil structure in both Q38 and P67C with same predicted solvent accessibility.

## Future directions and conclusion

**Fig. 2:**
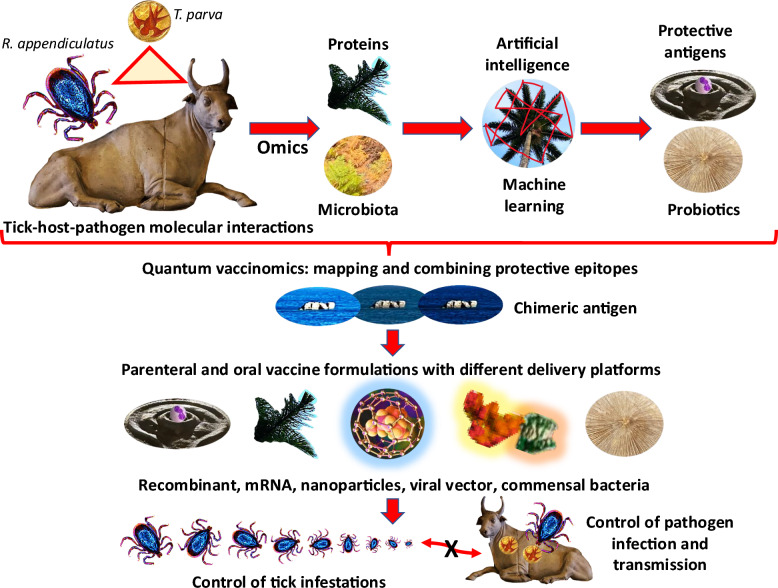
Future directions and novel vaccinology platforms and algorithms for designing and delivering vaccines against ticks and tick-borne diseases. To advance in vaccinology for the control of ticks and transmitted pathogens we should consider the characterization of tick-host–pathogen molecular interactions using omics analyses, application of metagenomics and metaproteomics analyses for the characterization of host and tick microbiome in response to pathogen infection, application of artificial intelligence (AI) and machine learning (ML) algorithms to omics dataset for the identification of candidate vaccine protective antigens and probiotics, design of chimeric antigens with the combination of protective epitopes using quantum vaccinomics approaches, and design and production of vaccine formulations using different delivery platforms with chimeric antigens and probiotics

On the basis of the limitations about the knowledge of the mechanisms involved in *T*. *parva* infection and host protective mechanisms and advances in vaccinology for designing effective vaccines for pathogen control, the characterization of host–pathogen and tick-pathogen molecular interactions will contribute to the identification of candidate protective antigens and commensal bacteria for designing combined vaccine formulations to control tick infestations and pathogen infection. To address these limitations, the proposed experimental approaches should consider (Fig. 2):

- Characterization of tick-host–pathogen molecular interactions using transcriptomics and proteomics analyses in host blood and tick tissues for the identification and characterization of host, tick, and pathogen proteins followed by analysis of interaction networks between these proteins. These analyses would lead to the identification and validation in vitro and in vivo of candidate proteins involved in pathogen infection in both ticks and cattle and the protective mechanisms in persistently infected cattle in the absence of detectable parasitemia and thus with activation of natural protective mechanisms.
- Characterization of host and tick microbiome in response to pathogen infection. Application of metagenomics and metaproteomics analyses will result in the identification and analysis of bacterial microbiota and the characterization of the effect of pathogen infection on both cattle and tick microbiota composition.
- Identification of candidate vaccine protective antigens and commensal bacteria such as *Lactobacillus* and *Pseudomonas* spp. Application of artificial intelligence (AI) Big Data analysis and machine learning (ML) algorithms for the identification of candidate protective antigens and commensal bacteria with high alpha-gal content for probiotic and/or paratransgenic interventions. Identified antigens should be evaluated for protective capacity alone and in combination with candidate probiotics in uninfected cattle naturally exposed to *T*. *parva*. Finally, mapping of reactive epitopes in selected tick and pathogen-derived antigens using sera from protected cattle will lead to the design of chimeric antigens with the combination of candidate protective epitopes using quantum vaccinomics approaches.
- Characterization using serum proteomics and transcriptomics of the host immune response to selected vaccine antigens and probiotic commensal bacteria with high alpha-gal content in single and combined formulations in experimental animal models (zebrafish and humanized alpha-gal synthase-KO mice alpha-gal negative organisms) and in cattle uninfected and naturally exposed to *T*. *parva*. Cattle produce alpha-gal but also generate antibodies against this biomolecule with protective capacity [39].
- Production of vaccine formulations using different delivery platforms (e.g., recombinant, mRNA, nanoparticles, viral vector) with chimeric antigens alone and in combination with candidate probiotic commensal bacteria for evaluation in cattle against *T*. *parva* infection and *R*. *appendiculatus* infestations. Vaccine formulations could be administered parentally or orally with heat-inactivated mycobacteria as immunostimulant [21].
- Evaluation of vaccine efficacy and effectiveness in controlled pen and field trials using a personalized vaccinology approach with international collaborations in developing countries with high incidence of tick-borne diseases [7, 40].

These approaches will advance in addressing gaps in the characterization of host–pathogen and tick-pathogen molecular interactions and the design and evaluation of novel vaccine formations for the control of ECF and other tick-borne diseases.

## Data Availability

No datasets were generated or analyzed during the current study.
